# Deep Learning for Delineation of the Spinal Canal in Whole-Body Diffusion-Weighted Imaging: Normalising Inter- and Intra-Patient Intensity Signal in Multi-Centre Datasets

**DOI:** 10.3390/bioengineering11020130

**Published:** 2024-01-29

**Authors:** Antonio Candito, Richard Holbrey, Ana Ribeiro, Christina Messiou, Nina Tunariu, Dow-Mu Koh, Matthew D. Blackledge

**Affiliations:** 1 Division of Radiotherapy and Imaging, The Institute of Cancer Research, London SM2 5NG, UKmatthew.blackledge@icr.ac.uk (M.D.B.); 2 Department of Radiology, The Royal Marsden NHS Foundation Trust, London SW3 6JJ, UK

**Keywords:** deep learning, segmentation, Whole-Body Diffusion-Weighted Imaging (WBDWI), metastatic bone disease, multiple myeloma

## Abstract

Background: Whole-Body Diffusion-Weighted Imaging (WBDWI) is an established technique for staging and evaluating treatment response in patients with multiple myeloma (MM) and advanced prostate cancer (APC). However, WBDWI scans show inter- and intra-patient intensity signal variability. This variability poses challenges in accurately quantifying bone disease, tracking changes over follow-up scans, and developing automated tools for bone lesion delineation. Here, we propose a novel automated pipeline for inter-station, inter-scan image signal standardisation on WBDWI that utilizes robust segmentation of the spinal canal through deep learning. Methods: We trained and validated a supervised 2D U-Net model to automatically delineate the spinal canal (both the spinal cord and surrounding cerebrospinal fluid, CSF) in an initial cohort of 40 patients who underwent WBDWI for treatment response evaluation (80 scans in total). Expert-validated contours were used as the target standard. The algorithm was further semi-quantitatively validated on four additional datasets (three internal, one external, 207 scans total) by comparing the distributions of average apparent diffusion coefficient (ADC) and volume of the spinal cord derived from a two-component Gaussian mixture model of segmented regions. Our pipeline subsequently standardises WBDWI signal intensity through two stages: (i) normalisation of signal between imaging stations within each patient through histogram equalisation of slices acquired on either side of the station gap, and (ii) inter-scan normalisation through histogram equalisation of the signal derived within segmented spinal canal regions. This approach was semi-quantitatively validated in all scans available to the study (N = 287). Results: The test dice score, precision, and recall of the spinal canal segmentation model were all above 0.87 when compared to manual delineation. The average ADC for the spinal cord (1.7 × 10^−3^ mm^2^/s) showed no significant difference from the manual contours. Furthermore, no significant differences were found between the average ADC values of the spinal cord across the additional four datasets. The signal-normalised, high-b-value images were visualised using a fixed contrast window level and demonstrated qualitatively better signal homogeneity across scans than scans that were not signal-normalised. Conclusion: Our proposed intensity signal WBDWI normalisation pipeline successfully harmonises intensity values across multi-centre cohorts. The computational time required is less than 10 s, preserving contrast-to-noise and signal-to-noise ratios in axial diffusion-weighted images. Importantly, no changes to the clinical MRI protocol are expected, and there is no need for additional reference MRI data or follow-up scans.

## 1. Introduction

WBDWI is an increasingly used non-invasive technique for detecting and assessing response to systemic treatments in patients with bone disease resulting from advanced prostate and breast cancer [1,2,3,4]. Furthermore, it is now recommended as first-line imaging for evaluation of multiple myeloma (MM) [5] due to its high sensitivity. The technique is generally well-tolerated by patients and facilitates long-term monitoring without radiation burden [6]. The technique offers excellent contrast between healthy and diseased bone that allows qualitative assessment “at a glance” of cancer spread across the skeleton; high-b-value images (>800 s/mm^2^) typically demonstrate hyper-intensity in regions of bone disease compared with background tissues. Acquisitions of images at two or more b-values also enable calculation of full-body maps of the Apparent Diffusion Coefficient (ADC), which may reflect tumour cellularity [7]. Evidence suggests that an increase in tumour ADC following treatment indicates a positive response [8,9].

Unfortunately, due to the influence of T1/T2 weighting, proton density, and coil sensitivity, it is difficult to compare signal intensities of acquired diffusion-weighted images throughout the course of treatment in an individual patient, or indeed compare signal intensities of images acquired from different scanners and/or vendors [10,11]. Furthermore, signal inhomogeneities across the imaging field of view (a “bias field”) and non-uniform signal between anatomical acquisition stations on WBDWI could degrade the accuracy of approaches for automatic tumour delineation of these datasets [12]. Signal normalisation techniques could improve clinician assessment of bone disease from WBDWI by improving lesion localisation, diameter measurement, and assessment of post-treatment changes by standardising the display of high-b-value images. Moreover, such techniques could facilitate the development of accurate automated tools for delineating bone diseases from high-b-value images [13,14], which would provide fast access to quantitative response biomarkers (ADC and lesion volume) in a deleterious disease for which there are no currently approved ways to assess early treatment changes. Ultimately, this could potentially reduce morbidity and improve survival rates [15].

Jager et al. [16] introduced a new method for intensity signal standardisation from MRI data using properties of all acquired images jointly (e.g., T1- and T2-weighted images). The method employed a non-rigid registration algorithm between the joint probability density distributions of a newly acquired image and a reference MRI dataset. However, we sought to develop a fast, WBDWI-only approach, as mis-registrations between diffusion-weighted imaging and other contrasts (caused by magnetic field inhomogeneities and the use of echo-planar imaging in WBDWI [17]) prohibit the use of joint histograms. Blackledge et al. [18] proposed an algorithm that might improve the standardisation and interpretation of WBDWI, which synthesises new contrast by combining voxel-wise ADC with voxel-wise estimates of the ADC uncertainty (thus removing the need for high-b-value images). The authors demonstrated initial results in a cohort of 16 patients with advanced prostate cancer (APC) but noted the limitation that protocols must include at least three b-values, which can make the approach difficult to adopt at some centres. Ceranka et al. [19] proposed a new method for signal intensity normalisation between baseline and follow-up whole-body MRI. The method involved two steps. (i) A 3D T1-weighted follow-up image was registered to the baseline image using B-spline deformable registration, and (ii) histogram matching was applied to the follow-up image to normalise signal intensity. The authors successfully applied this approach to a cohort of healthy volunteers and patients with APC (10 whole-body MRIs). The computational time of the registration step was 30 min, and no external validation datasets were used for testing.

Despite these recent advances, there remains a lack of clinical tools for inter-scan signal intensity normalisation based on WBDWI alone. We therefore aimed to develop an algorithm that met the following requirements: (i) fast computation time (within seconds), (ii) operates on WBDWI data alone, (iii) does not impact the underlying contrast-to-noise ratio (CNR) of images, and (iv) can be used on a single dataset with or without the availability of a follow-up scan. Our methodology is inspired by the approach taken by Padhani et al. [20], who normalised the signal intensity of high-b-value images by comparing against the signal intensity within muscle, kidney, and spinal cord signal following manual delineation of these regions. The authors derived the results from a cohort of 16 patients without metastatic bone disease, 21 patients with untreated metastases of breast cancer, and 12 patients with myeloma. However, their approach required manual delineation of anatomical regions, which is a significant barrier to clinical implementation of the technique. We have developed a fully automated workflow that leverages deep learning to automatically delineate the entire spinal canal on WBDWI and subsequently uses the pixel intensities within this region for histogram matching across cohort studies. 

## 2. Material and Methods

### 2.1. Patient Population and MRI Protocol

Initial training and testing of our spinal canal segmentation model was conducted in a cohort of 40 patients with confirmed APC (**Dataset (1)**); the training/test split for these patients was 32/8. A further “external” validation dataset comprised 85 patients with confirmed APC from three cohorts (**Dataset (2)** = 33 patients, **Dataset (3)** = 22 patients, and **Dataset (4)** = 18 patients), and 12 patients with confirmed diffuse MM from a single cohort (**Dataset (5)**). Patients with confirmed APC underwent baseline and follow-up WBDWI scans and patients with MM only had one WBDWI scan before treatment initiation. As a result, the total number of studies in the training, testing, and external validation cohorts were 64, 16, and 207, respectively. All images were acquired at a single imaging centre except **Dataset (3)** and 7 patients from **Dataset (2)**, which were performed at two additional imaging facilities.

WBDWI images were acquired using a 1.5 T scanner (MAGNETOM Aera/Avanto, Siemens Healthcare, Erlangen, Germany) across 4–5 anatomical stations (depending on patient height) from skull to mid-thigh (metastatic bone disease) or skull vertex to knees (myeloma) using two (50/900 s/mm^2^) or three (50/600/900 s/mm^2^) b-values. Each station comprised 40 slices with a slice thickness of 5–6 mm. Echo-planar image acquisition was used (GRAPPA parallel image acceleration R = 2), using a double-spin echo diffusion encoding scheme applied over three orthogonal encoding directions [21,22]. All MRI parameters are reported in Table 1 for each dataset involved in the study. All data were fully anonymised, and the study was performed in accordance with the Declaration of Helsinki (2013). A local ethical committee waived the requirement of patient consent for use of these retrospective datasets.

### 2.2. Spinal Canal Delineation

#### 2.2.1. Dataset Description

Patient data from **Dataset (1)** were split into training (24 patients, 48 studies), validation (8 patients, 16 studies), and test (8 patients, 16 studies) datasets. The segmentation model was trained using axial images extracted from all available WBDWI studies; thus, the total size of the training and validation data was 8749 and 2758 axial images, respectively, and the test data consisted of 2687 axial images. A medical physicist with over 2 years’ experience with WBDWI defined the ground truth by manually delineating the spinal cord and surrounding CSF for all WBDWI scans in **Dataset (1)** (80 studies in total). Accuracy of these delineations was confirmed by two radiologists, each with over 10 years’ experience in WBDWI.

#### 2.2.2. Image Pre-Processing

A deep learning model with U-Net architecture [23] was trained to automatically delineate the spinal cord and surrounding CSF on WBDWI scans. The network involves a 2-channel input: (i) the ADC map, and (ii) the estimated intercept (S0) image at b = 0 s/mm^2^. The ADC map and S0 image were derived by fitting a monoexponentially decaying model to the diffusion data [18,24] (negative ADC values were not removed from ADC maps so as not to introduce artificial boundaries in the derived images). The single-channel output of the deep learning model was the estimated segmentation of the spinal cord and surrounding CSF. All images were interpolated to matrix = 256 × 256 and resolution = 1.6 × 1.6 mm, and input images were normalised using the following transformations:

(1) $$scaled\;ADC\;map=ADC\;map\;/\;3.5\;10^{-3}{mm}^{2}/s$$

(2) $$scaled\;S0\;image=\log\left(S0\;image\right)\;/max(log(S0\;image))$$

#### 2.2.3. U-Net Model Architecture and Hyper-Parameter Selection

The U-Net architecture implemented in this study employed encoder (contractive) and decoder (expansive) symmetrical paths with skip connections at each level. The encoder path involved convolution blocks each followed by max pooling and dropout layers (dropout rate of 0.2), enabling the extraction of hierarchical features. Encoder convolution blocks consisted of two 2D convolutional layers with a 3 × 3 kernel (stride = 1), followed by batch normalisation and rectified linear unit (ReLU) activation. The encoder path featured four down-sampling steps with kernel filters of 32, 64, 128, and 256, reaching a bottleneck depth of 512. The decoder path used 2D transposed convolutions (kernel = 3 and stride = 2) for up-sampling and concatenating feature maps from the decoder path to capture fine-grained details. Additionally, dropout and two 2D convolutional layers followed the transposed operation at each level of the encoder path. Finally, the model generated a single-channel output representing the segmentation mask of the input image through a 1 × 1 convolutional layer with sigmoid activation applied to the output of the final decoder convolutional block. Dropout layers were incorporated to mitigate overfitting, while batch normalisation enhances model stability and convergence during training. Adam optimisation with an initial learning rate of 10^−3^ was used to minimise each loss function investigated over 150 epochs using a batch size of 8 slices. To facilitate efficient training and effective fine-tuning of the U-Net model, a dynamic learning rate strategy was implemented. This strategy involved reducing the learning rate by half when the loss function failed to improve for 10 consecutive epochs, continuing this process until the learning rate reached a minimum value of 10^−5^. A summary of the network hyper-parameters is reported in the Supplementary Material (Table S1). Four loss functions were compared to identify the best trade-off between precision and recall due to the significant imbalance between the number of voxels from the background image and the foreground spinal cord mask. Definitions of the losses investigated and their respective hyper-parameters are shown in Table 2. Where applicable, loss function hyper-parameters were optimised by identifying values that minimised the validation Dice score (1-Dice loss). All algorithms were implemented in python (v.3.7) using Tensorflow v.2.3.1 and Keras toolboxes, running on a Windows platform (v.10.0.19) accelerated by an NVIDIA RTX6000 GPU 24 GB RAM (Santa Clara, CA, USA).

#### 2.2.4. Post-Processing

The final layer of the 2D U-Net model was a sigmoid activation function, which generates a value ranging from 0 (background) to 1 (spinal cord and surrounding CSF). For an individual patient study, these values were linearly interpolated between adjacent slices to create a final 3D prediction from the most superior slice to the most inferior. Subsequently, a threshold of 0.5 was applied to derive the final binary mask. Once the spinal canal was delineated, the predicted segmentations were transferred to calculated ADC maps; the resulting ADC values were modelled using a 2-component Gaussian Mixture Model (GMM) to reflect values in the spinal cord and surrounding CSF, respectively [29]. The GMM model was implemented using the Scikit-Learn v.0.23.2 software package. 

### 2.3. WBDWI Signal Normalisation

**Figure 1:**
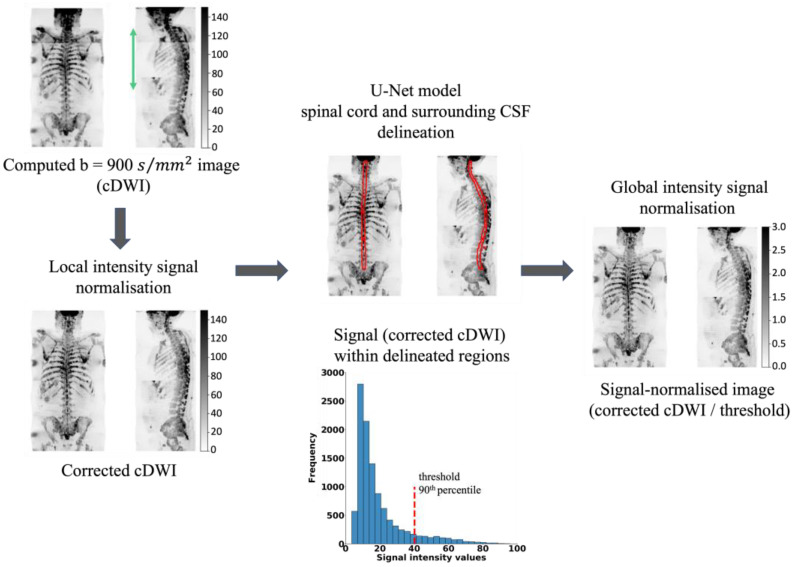
Schematic representation of the developed method for intensity signal normalisation on WBDWI. The method involves (i) computing the b = 900 s/mm^2^ image (cDWI) from the derived ADC map and S0 image and composing the whole-body cDWI, (ii) correcting the signal inhomogeneity (green arrow) across sequential stations, (iii) deriving the spinal cord and surrounding CSF segmentation from the developed U-Net model, (iv) generating the “signal-normalised image” by dividing the entire image by the 90th percentile of cDWI (step (ii)) signal within the spinal cord and surrounding CSF region.

Our signal normalisation pathway consists of three core steps, as illustrated in Figure 1:

(1) A whole-body *b_c_* = 900 s/mm^2^ image is computed (cDWI) to optimize the SNR and increase the suppression of the background signal for the detection of bone metastases [24]. The cDWI images are computed from the estimated *S*_0_ images and ADC maps for each station using:
  (3) $$S\left(b_{c}\right)=S_{0}e^{-b_{c}\;\cdot \;ADC}$$
(2) The signal intensity of sequential stations of derived cDWI volumes is normalised by applying a linear scaling term to the cDWI data that minimises the mean square error between cumulative frequency curves of cDWI intensities from axial images on either side of each station boundary, as previously described [30,31].
(3) The spinal cord and surrounding CSF segmentations derived using the U-Net model (Section 2.2) are transferred to the cDWI images and the voxel values across the entire field of view are standardised to the 90th percentile of the signal within the entire spinal canal.

### 2.4. Evaluation Criteria

#### 2.4.1. Spinal Canal Segmentation

To assess the best set of hyper-parameters and loss function for the U-Net model, patient-wise Dice score, precision, and recall between the manual and derived spinal cord and surrounding CSF masks were reported for all validation patients from **Dataset (1)**. Volume and average cross-sectional area of the derived spinal canal segmentations were reported together with the true values for all patients in the validation and test datasets of **Dataset (1)**, along with parameters estimated from GMM modelling of the ADC values. Significant differences in the performance of the U-Net model and manual delineations were assessed using a Wilcoxon signed-rank test (*p* < 0.05 indicating significance). 

A qualitative visual assessment of segmentations derived from the U-Net model was performed on the external validation datasets for which no ground-truth contours were available (**Datasets (2)**, **(3)**, **(4)**, and **(5)**). Derived spinal cord and CSF segmentations were superimposed on coronal and sagittal maximum-intensity projections (MIPs) of the high-b-value (b = 900 s/mm^2^) images to facilitate the visual assessment of delineated regions. Furthermore, ADC distributions and volumes within the spinal cord and surrounding CSF (obtained following GMM modelling) were compared with those obtained in the test/validation data from **Dataset (1)** to provide a semi-quantitative evaluation of segmentation accuracy. Differences between the derived spinal cord or CSF volumes across the external validation datasets were assessed using the ANOVA test (*p* < 0.05 indicating significance). Average ADC values of the spinal cord and surrounding CSF for each external validation dataset, as derived using the GMM, were assessed for significant differences using the Wilcoxon signed-rank test (*p* < 0.05 indicating significance). 

#### 2.4.2. Intensity Signal WBDWI Normalisation 

A qualitative assessment of the developed intensity signal normalisation pipeline was performed by comparing visual appearance of derived images with the standard b = 900 s/mm^2^ images for all patients in the test images from **Dataset (1)**, and all external validation datasets, using fixed window settings.

A semi-quantitative assessment was performed by an experienced radiologist with 10+ years of experience in WBDWI who delineated regions of suspect bone disease according to MET-RADS-P guidelines [1] in 10 patients from **Dataset (2)** (both baseline and post-treatment scans). Resulting regions of interest were transferred to the signal-normalised and conventional b = 900 s/mm^2^ images to derive the voxel-wise distribution of signal intensity within the delineated regions. Differences in the tumour distributions of normalised and conventional image signals were visually compared between patients and between successive scans of the same patient. The number of bone metastases delineated on average per WBDWI scan was 15 with an average total disease volume of 430 mL. 

## 3. Results

### 3.1. Spinal Canal Segmentation

The best-performing U-Net model demonstrated an average validation dice score of 0.871 when trained using the Focal Tversky loss function, as shown in Table 3. This loss function showed the best trade-off between precision and recall by tuning the coefficients *α* = 0.7, *β* = 0.3 (weighting more for false negative than false positive detection) and improving small region contribution to the loss by tuning *γ* = 1.1. The average dice score, precision, and recall between manual and derived segmentations from the best-performing U-Net model across all hold-out test datasets were 0.879, 0.869, and 0.896, respectively. 

**Figure 2:**
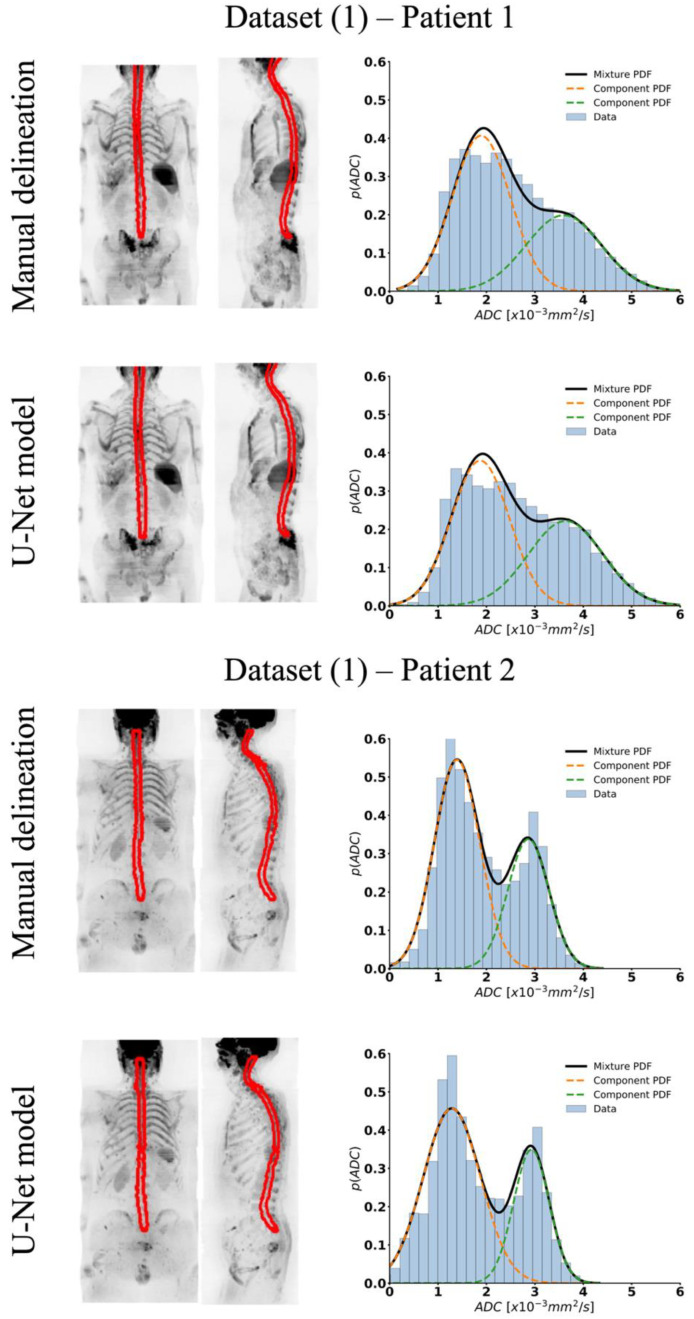
Coronal and sagittal view of maximum-intensity projection (MIP) of b = 900 s/mm^2^ image and superimposed manual and derived spinal cord and surrounding CSF segmentation for two test datasets (**Dataset (1)**). Histogram of ADC values within manual and automated (U-Net model) segmentations and fitting using a 2-component GMM for the same test datasets.

The U-Net model generated spinal cord and surrounding CSF segmentations that show excellent agreement with the true labels and patient anatomy without overfitting on the test datasets, as shown in Figure 2. Furthermore, ADC histograms within the segmented regions illustrate two peaks for both methods. A similar result was observed in all other test data, supporting our hypothesis for the use of a two-component GMM for classifying pixels as either belonging to the spinal cord or CSF.

Shape-based and GMM parameters from our U-Net model were compared with manually defined contours for both validation and test data from **Dataset (1)** and are reported in Table 4. Median measure of volume and average cross-section area for the validation data were 169 mL and 179 mm^2^, respectively, and 164 mL and 204 mm^2^ for the test data, as derived using our automated model. No significant differences were observed between manual and automated segmentation. Parameters derived from the GMM showed similar trends for both methods and again without any significant differences.

**Figure 3:**
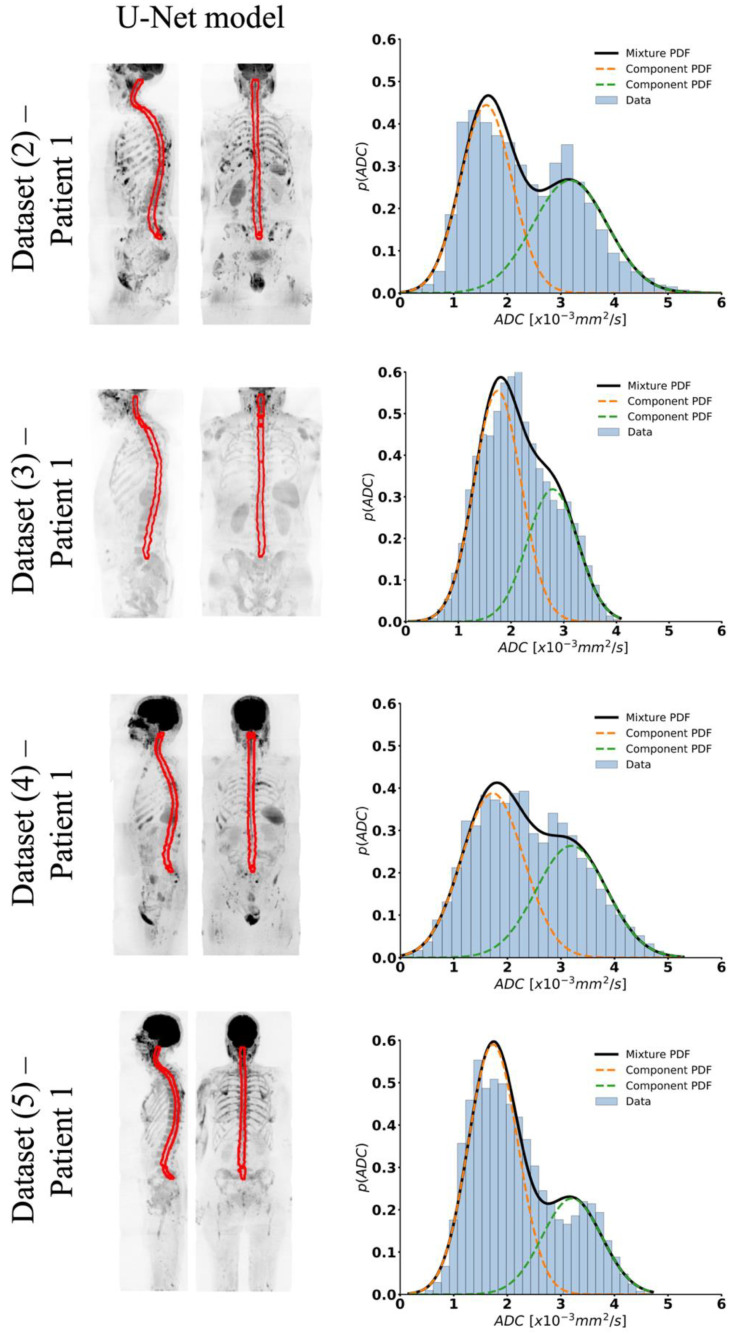
Coronal and sagittal view of maximum-intensity projection (MIP) of b = 900 s/mm^2^ image and superimposed derived spinal cord and surrounding CSF segmentation for four patients in the external WBDWI validation datasets. Histogram of ADC values within automated (U-Net model) segmentations and fitting using a 2-component GMM for the same external validation datasets.

Figure 3 illustrates four example patients, each randomly selected from the external validation **Datasets (2)–(5)**. Visually, our segmentation model correctly predicted the position of the spinal cord within each patient, without showing signs of overfitting. Moreover, the derived ADC histogram within the delineated regions predominantly showed the same bimodal distribution observed in the training data. 

**Figure 4:**
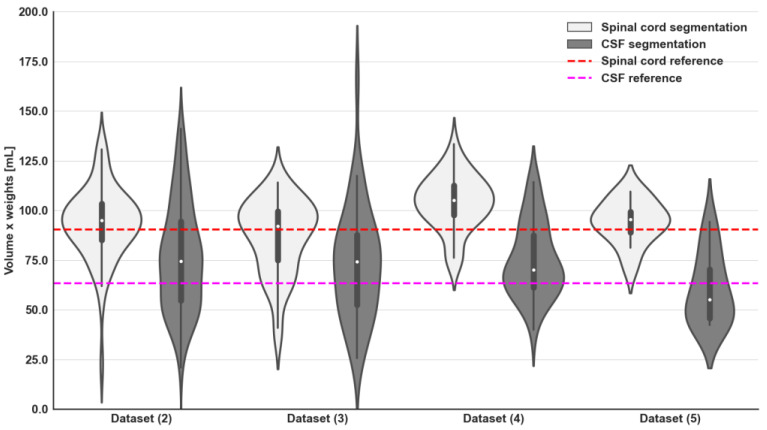
Violin plots of the derived spinal cord and CSF volumes (weight per segmentation volume) from U-Net model segmentations and GMM fitting of ADC values within the delineated regions for all external validation datasets. Additionally, reference values for spinal cord (indicated by the red dashed line) and CSF (indicated by the purple dashed line) volumes were obtained from manual contours of the spinal canal on WBDWI studies included in **Dataset (1)**. No statistical differences were observed for CSF volumes across different external validation datasets (the ANOVA test showed a *p*-value > 0.05). The same trend was observed for the spinal cord volumes, if **Dataset (4)** is excluded.

**Figure 5:**
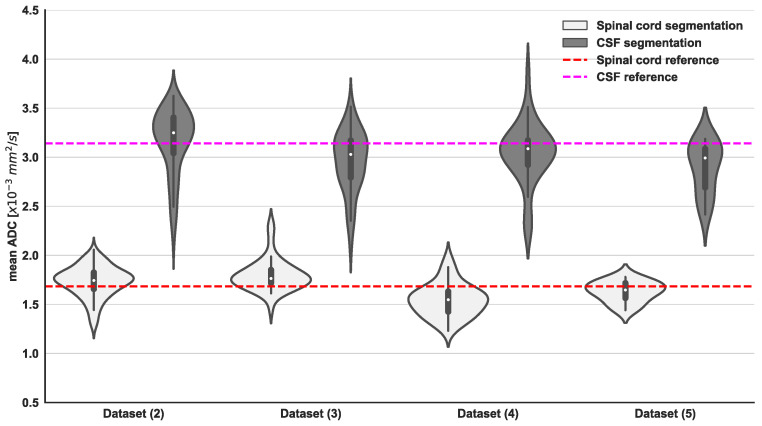
Violin plots of derived spinal cord and CSF ADC mean from U-Net model segmentations and GMM fitting of ADC values within the delineated regions for all external validation datasets. Additionally, reference values for spinal cord (indicated by the red dashed line) and CSF (indicated by the purple dashed line) ADC mean were obtained from manual contours of the spinal canal on WBDWI studies included in **Dataset (1)**. Statistical differences were observed between the 2 components of the GMM from the same dataset (Wilcoxon signed-rank test showed a *p*-value < 0.05). The same trend was observed for all the external validation datasets.

The distributions of volumes and average ADC for the spinal cord and CSF are presented in Figure 4 and Figure 5, respectively. No statistical differences were observed in these distributions between the different datasets used (*p* = 0.13), with exclusion of **Dataset (4)**, which demonstrated a marginally increased volume of the spinal cord. Derived volumes for the spinal cord and CSF across different datasets range from 92 to 105 mL and from 55 to 74 mL, respectively. Furthermore, derived values of ADC mean for spinal cord and CSF were on average 1.7 and 3.2 × 10^−3^ mm^2^/s, respectively, with significant differences between both components (*p* < 0.001).

### 3.2. Intensity Signal WBDWI Normalisation 

**Figure 6:**
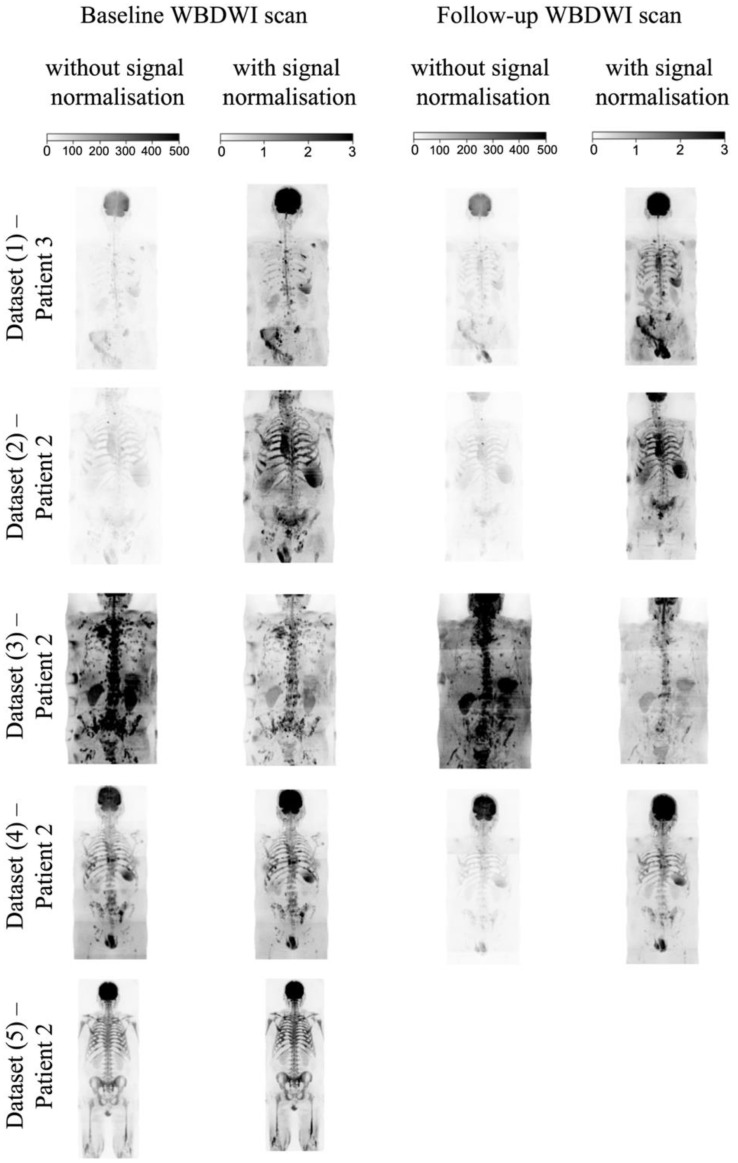
Coronal view of maximum-intensity projection (MIP) of b = 900 s/mm^2^ image with and without intensity signal normalisation for baseline and follow-up WBDWI scans of a patient from the test dataset and for four patients from the external validation datasets. Images with and without signal-based normalisation were scaled using a fixed window of 0–500 and 0–3, respectively. Signal-normalised images show a more uniform signal across sequential stations and no variation in intensity values in skeleton areas with the same properties as bone lesions across all datasets. In contrast, acquired b = 900 s/mm^2^ images show signal inhomogeneity across sequential stations, between baseline and follow-up scans and different WBDWI datasets.

Examples of normalised high-b-value MIP images are compared with acquired b = 900 s/mm^2^ MIP images in Figure 6. MIPs of both high-b-value and signal-normalised images for patients with APC and MM show high signal intensity for suspected metastatic bone lesions across much of the skeleton. For the normalised images, however, it is straightforward to scale the intensity level using a fixed window level = 1.5 and window width = 3.0. By contrast, the acquired high-b-value images demonstrate significant signal variation between and within datasets when presented using fixed window level = 250 and window width = 500. Our pipeline generates the normalised high-b-value images in seconds and does not require spatial registration of successive time-points of individual patients.

**Figure 7:**
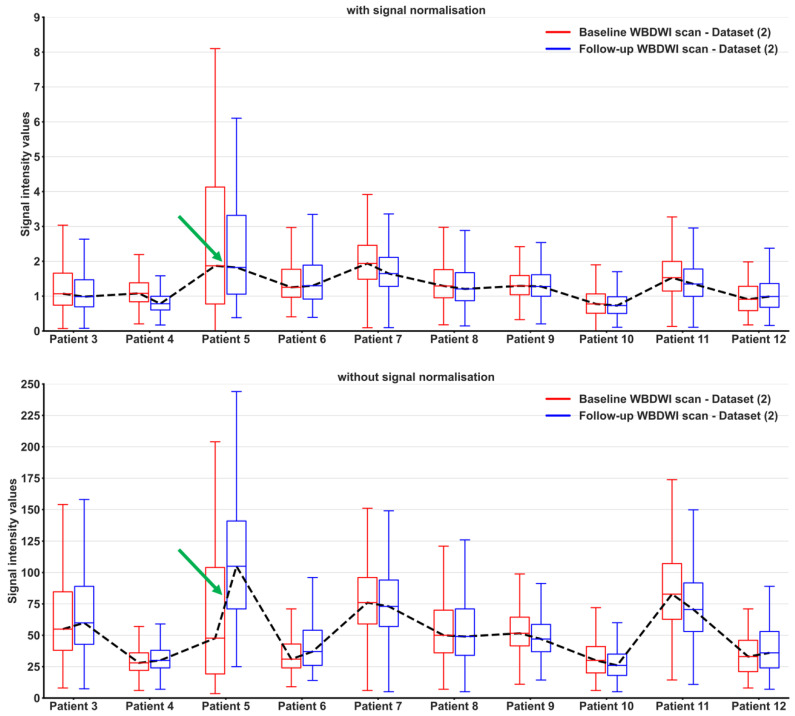
Voxel-wise distribution of signal intensity values within delineated bone metastases from 10 patients with APC who underwent baseline and WBDWI scans. Signal intensity values are compared between our signal-normalised images (**top**) and conventional b = 900 s/mm^2^ images (**bottom**). Our pipeline for normalising signal intensity on high-b-value WBDWI datasets reduces inter-patient variation in image signal within delineated lesions. Furthermore, we observe reduced variation in lesion signal intensity between scans for the same patient using our approach (see Patient 5 for a particularly poor example, green arrow).

As presented in Figure 7, distributions of tumour signal intensity values from normalised images were more homogenous between patients and between successive scans of the same patient (signal range of tumours 0.65–1.25), when compared with conventional high-b-value signal intensities. This could improve radiological interpretation of scans and provide a range of standardised signal intensities for regions of suspected disease on WBDWI. 

## 4. Discussion and Conclusions

Inter- and intra-patient signal normalisation on WBDWI is a challenging task but could vastly improve the consistency of interpretation of high-b-value imaging and facilitate the development of automated tumour delineation techniques. In this article, a novel method has been developed to improve signal normalisation of WBDWI, using a validated deep learning approach for automatic delineation of the spinal canal from which signal statistics can be much more readily derived.

The dice scores between manual and automated spinal canal segmentations were excellent in all cases tested (>0.87) and demonstrated visually exceptional results when qualitatively assessed in four external validation datasets (85 patients, 207 scans). The segmentation masks, superimposed on coronal and sagittal MIPs of high-b-value images, visibly matched the patient anatomy in all cases, even in those data acquired by external imaging centres with variations in MRI scanning protocol. 

A further advantage of this technique is that segmentations may be transferred to the ADC map to interrogate distributions of ADC within delineated regions. We utilised a two-component GMM of the ADC distribution to derive separate sub-segmentations for the spinal cord and surrounding CSF. Parameters estimated from such models demonstrated no significant differences between manual and automated segmentation in 16 validation/test patients (32 studies in total). Likewise, a bimodal ADC distribution within delineated regions and consistent spinal cord/CSF volumes and average ADC values were derived for patients in the external validation datasets.

Several studies by other investigators have also explored deep learning to automatically delineate the spinal cord from MRI data. Lemay et al. [32] introduced a 3D patch-based U-Net model, which successfully delineated a bounding box for the spinal canal from T2w MRI images (this model served as a preliminary step for a second algorithm focused on detecting suspected spinal cord tumours). They achieved a dice score of 0.89 on a single-centre test cohort. McCoy et al. [33] proposed a 2D U-Net model for automated delineation of the spinal canal from axial T2w images acquired using a 3T MRI, facilitating the detection of acute spinal cord injuries. The model achieved a dice score above 0.9 on 560 axial images from a single-centre test cohort. Gros et al. [34] developed a cascade of three Convolutional Neural Networks (CNNs) for spinal cord centre line detection, delineation of the spinal cord, and identification of suspected Multiple Sclerosis (MS) lesions. The authors employed a 2D patch-based U-Net model with dilated kernels in the decoder path for spinal cord centre line detection from T1w and T2w MRI images, followed by a 3D patch-based U-Net model with prior knowledge of the spinal cord location for spinal canal segmentation. The dice score between manual expert and automated methods was above 0.9 from a multi-centre cohort of 1042 patients. Remarkably, our method demonstrates segmentation accuracies close to those reported by these studies, yet operates on diffusion-weighted images alone, which are typically acquired with lower resolution than T1 and/or T2 weighted imaging. Furthermore, WBDWI can be severely affected by geometric distortions, which warrants the need for a dedicated WBDWI approach; as far as we are aware, we are the first to propose such a methodology.

Intensity signal normalisation was compared against the original acquired high-b-value images on test and external WBDWI scans. Our approach greatly improved harmonisation of signal intensity across sequential stations within each patient, and between patients/institutions. In future, this should facilitate more consistent clinical reading of WBDWI scans due to more uniform signal presentation and increase confidence in the results, even for less experienced scanning centres (though this will require further testing in larger-cohort, multi-centre studies). Global signal normalisation may also improve comparison of findings from the same patient at different time-points throughout the course of their treatment, thus providing clearer indications of disease changes. As our method requires only the b-value images from a single acquisition as input and does not affect the contrast-to-noise and signal-to-noise ratios in axial diffusion-weighted images, it may be readily applied to correct for inter-patient signal variation and/or intra-patient variation observed at different scanning dates/times for a single patient.

Recent publications have developed techniques for full skeleton segmentation on WBDWI [35,36,37]. By leveraging the results found in this study, it may be possible to directly utilise WBDWI signal within statistical or deep learning approaches to the segmentation of disease within the skeleton. Once defined, the derived ROIs can be transferred to the calculated ADC maps to derive biomarkers such as the Total Diffusion Volume (TDV), which reflects the estimated tumour burden within the skeleton, and median global ADC. Existing evidence points to these as emergent biomarkers of response for metastatic bone disease treated with novel therapeutics in patients with APC [38].

One of the limitations of this study was the smaller number of datasets used for external validation that were acquired at different centres; **Dataset (2)** and **Dataset (3)** included seven and twenty-two patients acquired at different imaging centres, respectively. However, it is worth noting that all patients underwent multiple post-treatment scans, thereby enhancing the impact of these datasets. Furthermore, the supervised deep learning model for automatically delineating the spinal canal from WBDWI has been developed and tested using labels provided by a single annotator. To address concerns about potential bias, the segmentation accuracy in external validation WBDWI datasets was assessed through qualitative evaluation by two radiologists in functional cancer imaging, each with over 10 years of experience. This evaluation involved overlaying the spinal canal masks on ADC maps and S0 images, ensuring that the boundaries delineated by the automated tool aligned with the true shape and position of the spinal canal visible in WBDWI scans. Furthermore, the evaluation of spinal canal segmentation accuracy and image normalisation on external validation datasets was either qualitative or semi-quantitative in nature. Prospective evaluation of our tool in a multi-centre setting is the subject of a currently ongoing trial that is evaluating the effectiveness of these tools for automatic delineation of bone disease from WBDWI. 

In conclusion, our automated pipeline accurately delineates the spinal cord and surrounding CSF, which in turn can be used to normalise signals within and between WBDWI acquisitions. This could drastically improve visual assessment of disease in longitudinal WBDWI studies and thus has the potential to positively impact the healthcare of patients with advanced cancers. 

## Figures and Tables

**Table 1:** Scanning protocol and MRI parameters for all WBDWI datasets investigated in our study. Minimum and maximum values are displayed in parenthesis.

	APC Cohort Dataset (1) (40 Patients; 80 WBDWI Scans)	APC Cohort Dataset (2) (33 Patients; 115 WBDWI Scans)	APC Cohort Dataset (3) (22 Patients; 44 WBDWI Scans)	APC Cohort Dataset (4) (18 Patients; 36 WBDWI Scans)	MM Cohort Dataset (5) (12 Patients; 12 WBDWI Scans)
**MR scanner**	1.5T Siemens Aera	1.5T Siemens Aera/Avanto	1.5T Siemens Aera	1.5T Siemens Aera	1.5T Siemens Avanto
**Sequence**	Diffusion-Weighted SS-EPI	Diffusion-Weighted SS-EPI	Diffusion-Weighted SS-EPI	Diffusion-Weighted SS-EPI	Diffusion-Weighted SS-EPI
**Acquisition plane**	Axial	Axial	Axial	Axial	Axial
**Breathing mode**	Free breathing	Free breathing	Free breathing	Free breathing	Free breathing
**b-values [s/mm^2^]**	b50/b600/b900 for all patients	b50/b600/b900 for all patients	b50/b900 for 7 patients; B50/b600/b900 for 15 patients	b50/b600/b900 for all patients	b50/b900 for 9 patients; B50/b600/b900 for 3 patients
**Number of averages** **(per b-value)**	(2,2,4)–(3,3,5)	(3,3,5)	(3,5)–(3,3,5)	(3,6,6)	(4,4)–(2,2,4)
**Reconstructed resolution [mm^2^]**	[1.56 × 1.56–1.68 × 1.68]	[1.68 × 1.68–2.5 × 2.5]	[1.68 × 1.68–3.12 × 3.12]	[3.21 × 3.21]	[1.54 × 1.54–1.68 × 1.68]
**Slice thickness [mm]**	5	5	6	5	5
**Repetition time [ms]**	[6150–12,700]	[5490–10,700]	12,003	6320	[6150–14,500]
**Echo time [ms]**	[60–79]	69	[69–72]	76	[66.4–69.6]
**Inversion time** **(STIR fat suppression) [ms]**	180	180	180	180	180
**Flip angle [°]**	90	90	90	90	180
**Encoding code**	3-scan Trace	3-scan Trace	3-scan Trace	3-scan Trace	3-scan Trace
**Field of view [mm]**	[98 × 128–256 × 256]	[130 × 160–208 × 256]	[208 × 256–216 × 257]	[108–134]	[208 × 256–224 × 280]
**Receive bandwidth [Hz/Px]**	[1955–2330]	1955	1955	2195	[1984–2330]

**Table 2:** Definitions of the examined losses along with their corresponding hyper-parameters, which were used in the process of training the 2D U-Net model for automatically delineating the spinal canal from WBDWI. *TP* = true positives, *FP* = false positives, *FN* = false negatives, *y_n_* = true label for voxel *n* (0 = background, 1 = spinal canal), ${\hat{y}}_{n}$ = model-predicted label probability for voxel *n*.

Loss Name	Definition	Discussion
**Log-cosh Dice**	$LCDL=ln(cosh\left(DL\right))$ $\mathrm{where}\;DL=1-\frac{2\cdot TP}{2\cdot TP+FP+FN}$	This univariate transformation of the Dice loss, DL, has been suggested for improving medical image segmentation in the context of imbalanced distributions of labels [25].
**Combo**	$CL=DL-\omega \frac{1}{N}\sum_{n=1}^{N}y_{n}\cdot ln{\hat{y}}_{n}$ $+\left(1-y_{n}\right)\cdot ln\left(1-{\hat{y}}_{n}\right)$	A weighted sum of Dice and binary cross-entropy losses [26]. To identify the optimal weight ω∈(0,1) between these two losses, training/validation of the U-Net model was compared using values of ω from 0 to 1 at increments of 0.1.
**Tversky**	$TL=1-\frac{TP}{TP+\alpha \cdot FP+\beta \cdot FN}$	A generalised version of the Dice loss (α=β=0.5), this loss provides more nuanced balancing between a requirement for high sensitivity (α>β) or precision (α<β). The best trade-off was investigated by varying the values of *α* and *β*, from 0 to 1 with an increment of 0.1 [27].
**Focal Tversky**	$FTL={TL}^{\gamma }$	A further generalisation of the Tversky loss, this loss employs a third parameter *γ*, which controls the non-linearity of the loss. In class-imbalanced data, small-scale segmentations might result in a high TL score; however, *γ* > 0 causes a higher gradient loss, forcing the model to focus on harder examples (small regions of interest that do not contribute to the loss significantly) [28]. We varied *γ* from 1 to 3 with an increment of 0.1 to determine the optimal value.

**Table 3:** Mean and standard deviation of similarity metrics between manual and U-Net model automated segmentations of spinal canal for patients in **Dataset (1)** (for optimum hyper-parameters).

Loss Function	Dice Score	Precision	Recall
**Log cosh Dice**	0.865 ± 0.04	0.898 ± 0.03	0.839 ± 0.08
**Combo** (*ω* = 0.8)	0.858 ± 0.06	0.912 ± 0.02	0.819 ± 0.104
**Tversky** (*α* = 0.7, *β* = 0.3)	0.860 ± 0.04	0.844 ± 0.03	0.883 ± 0.08
**Focal Tversky** (*α* = 0.7, *β* = 0.3, *γ* = 1.1)	**0.871 ± 0.04**	**0.870 ± 0.03**	**0.878 ± 0.081**

**Table 4:** Median and interquartile range of volume and average cross-section area derived from manual and automated (U-Net model) segmentation of the spinal cord and surrounding CSF for all patients in the validation and test WBDWI datasets (**Dataset (1)**). Mean and standard deviations of parameters from fitting the ADC values within manual and automated (U-Net model) segmentation of the spinal cord and surrounding CSF using a 2-component GMM for all patients in the validation and test WBDWI datasets (**Dataset (1)**). Significant differences between manual and automated segmentation are for *p* < 0.05 (Wilcoxon paired rank-sum test: two-tailed).

	Volume [mL]	Average Cross-Section Area [mm^2^]	GMM—Weights		GMM—Means [×10^−3^ mm^2^/s]		GMM—Variance	
**Validation set** **(8 patients; 16 WBDWI scans)**			1^st^ comp PDF (spinal cord)	2^nd^ comp PDF (CSF)	1^st^ comp PDF (spinal cord)	2^nd^ comp PDF (CSF)	1^st^ comp PDF (spinal cord)	2^nd^ comp PDF (CSF)
**Manual delineation**	152 [138–188]	176 [151–184]	0.58 ± 0.09	0.41 ± 0.09	1.67 ± 0.20	3.17 ± 0.31	0.38 ± 0.09	0.59 ± 0.11
**U-Net model**	169 [141–192]	17 9 [158–192]	0.61 ± 0.08	0.40 ± 0.08	1.72 ± 0.13	3.17 ± 0.27	0.37 ± 0.1	0.58 ± 0.12
***p*-value**	0.91	0.94	0.31	0.31	0.04	0.12	0.69	0.16
	**Volume ** **[mL]**	**Average Cross-Section Area [mm^2^]**	**GMM—Weights**		**GMM—Means** **[×10^−3^ mm^2^/s]**		**GMM—Variance**	
**Holdout set (8 patients;** **16 WBDWI scans)**			1^st^ comp PDF (spinal cord)	2^nd^ comp PDF (CSF)	1^st^ comp PDF (spinal cord)	2^nd^ comp PDF (CSF)	1^st^ comp PDF (spinal cord)	2^nd^ comp PDF (CSF)
**Manual delineation**	160 [147–184]	199 [184–226]	0.59 ± 0.06	0.41 ± 0.06	1.70 ± 0.15	3.33 ± 0.28	0.35 ± 0.07	0.56 ± 0.20
**U-Net model**	164 [153–175]	204 [192–213]	0.60 ± 0.06	0.40 ± 0.06	1.73 ± 0.13	3.36 ± 0.28	0.35 ± 0.10	0.60 ± 0.19
***p*-value**	0.26	0.28	0.67	0.67	0.14	0.30	0.73	0.04

## Data Availability

Data can be shared upon request from the authors. However, this is subject to the establishment of an appropriate data-sharing agreement, given the sensitive nature of patient information involved.

## Supplementary Materials

The following supporting information can be downloaded at: https://www.mdpi.com/article/10.3390/bioengineering11020130/s1, Table S1: Training parameters of the spinal canal segmentation model.
