# Supplementary material for: Deep Learning for Delineation of the Spinal Canal in Whole-Body Diffusion-Weighted Imaging: Normalising Inter- and Intra-Patient Intensity Signal in Multi-Centre Datasets
Source: Bioengineering (Basel). 2024 Jan 29;11(2):130. doi: 10.3390/bioengineering11020130 (PMC10885936; doi:10.3390/bioengineering11020130)
Supplement: Supplementary file 1 [file bioengineering-11-00130-s001.zip › bioengineering-2723218 Supplementary Table S1.pdf]

Table S1. Training parameters of the spinal canal segmentation model.

Parameters	2D U-Net model for spinal canal segmentation
Input	S0 image + ADC map
Convolutional blocks for encoder and decoder paths	4
Kernel filters in the first convolutional block	32
Encoder convolutional block	Two convolutional layers with kernel size 3x3 and stride 1 followed by batch normalization and ReLU
Max pooling	For memory efficiency by reducing the spatial resolution of the feature maps
Dropout rate	0.2
Bottleneck layer depth	512
Decoder convolutional block	2D transposed convolution layers with kernel size 3x3 and stride 2 followed by dropout and two convolutional layers
Final layer	Sigmoid
Loss	Focal Tversky loss with $\alpha = 0.3$ $\beta = 0.7$ $\gamma = 1.1$
Optimizer	Adam
Dynamic learning rate	Start from 10^{-3} and decrease by half if the loss does not not improve for 10 consecutive epochs until a minimum value of 10^{-5}
Number of epochs	150
Batch size	8 axial images (256x256)
